# Histo-anatomical structure of the living isolated rat heart in two contraction states assessed by diffusion tensor MRI

**DOI:** 10.1016/j.pbiomolbio.2012.07.014

**Published:** 2012-10

**Authors:** Patrick W. Hales, Jürgen E. Schneider, Rebecca A.B. Burton, Benjamin J. Wright, Christian Bollensdorff, Peter Kohl

**Affiliations:** a Department of Cardiovascular Medicine, University of Oxford, Oxford OX3 7BN, UK; b National Heart and Lung Institute, Imperial College London, London UB9 6JH, UK; c Department of Physiology, Anatomy and Genetics, University of Oxford, Oxford OX1 3PT, UK; d Wellcome Trust Centre for Human Genetics, University of Oxford, Oxford OX3 7BN, UK; e Department of Computer Science, University of Oxford, Oxford OX1 3QD, UK

**Keywords:** Cardiac magnetic resonance imaging, Cardiac contraction, Myocardial histo-architecture, Diffusion tensor imaging, Myolaminae, 2D, two-dimensional, 3D, three-dimensional, ADC, apparent diffusion coefficient, CF, circumferential fibre, DTI, diffusion tensor imaging, FA, fractional anisotropy, FOV, field of view, IDL, interactive data language, LHF/RHF, left/right-handed helical fibre, LV, left ventricle, MRI, magnetic resonance imaging, NMR, nuclear magnetic resonance

## Abstract

Deformation and wall-thickening of ventricular myocardium are essential for cardiac pump function. However, insight into the histo-anatomical basis for cardiac tissue re-arrangement during contraction is limited. In this report, we describe dynamic changes in regionally prevailing cardiomyocyte (fibre) and myolaminar (sheet) orientations, using Diffusion Tensor Imaging (DTI) of ventricles in the same living heart in two different mechanical states. Hearts, isolated from Sprague–Dawley rats, were Langendorff-perfused and imaged, initially in their slack state during cardioplegic arrest, then during lithium-induced contracture. Regional fibre- and sheet-orientations were derived from DTI-data on a voxel-wise basis. Contraction was accompanied with a decrease in left-handed helical fibres (handedness relative to the baso-apical direction) in basal, equatorial, and apical sub-epicardium (by 14.0%, 17.3%, 15.8% respectively; *p* < 0.001), and an increase in right-handed helical fibres of the sub-endocardium (by 11.0%, 12.1% and 16.1%, respectively; *p* < 0.001). Two predominant sheet-populations were observed, with sheet-angles of either positive (*β*+) or negative (*β*−) polarity relative to a ‘chamber-horizontal plane’ (defined as normal to the left ventricular long-axis). In contracture, mean ‘intersection’-angle (geometrically quantifiable intersection of sheet-angle projections) between *β*+ and *β*− sheet-populations increased from 86.2 ± 5.5° (slack) to 108.3 ± 5.4° (*p* < 0.001). Subsequent high-resolution DTI of fixed myocardium, and histological sectioning, reconfirmed the existence of alternating sheet-plane populations. Our results suggest that myocardial tissue layers in alternating sheet-populations align into a more chamber-horizontal orientation during contraction. This re-arrangement occurs via an accordion-like mechanism that, combined with inter-sheet slippage, can significantly contribute to ventricular deformation, including wall-thickening in a predominantly centripetal direction and baso-apical shortening.

## 1 Introduction

Detailed knowledge of the histo-anatomy of the heart is crucial for understanding its mechanical (Waldman et al., 1988) and electrical (Kanai and Salama, 1995) behaviour. Myocardial tissue structure is intimately linked to heart function, and both change considerably in cardiac disease. However, a complete and consistent description of cardiac structure, and its deformation during the heartbeat, is lacking, largely due to the complex architecture of myocardial tissue, and the destructive techniques which traditionally have been used to examine it.

**Fig. 1:**
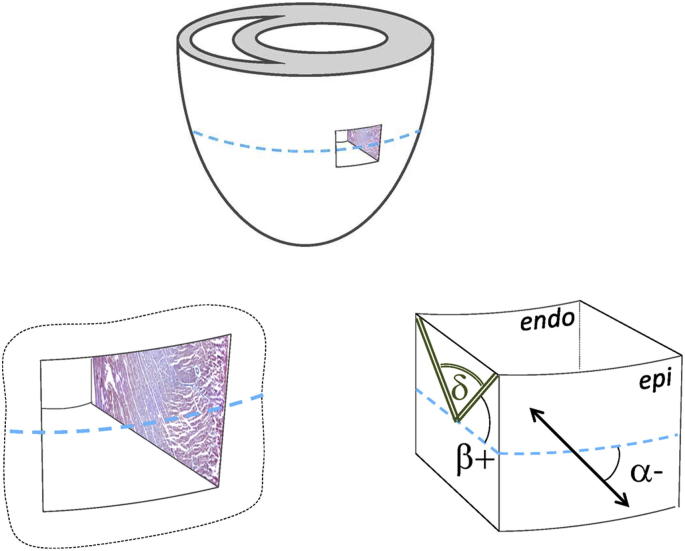
**Schematic illustration to introduce fibre- and sheet-angle definitions**. *Top*: conceptualization of the orientation of a ‘transmural tissue block’, removed from the left-ventricular (LV) free-wall, with reference to the heart's chamber-horizontal plane (blue dashed line). *Bottom-Left*: schematic representation of transmural cut, with indication of long-section tissue appearance (see Fig. 6 for original transmural long-section histology). *Bottom-Right*: excised transmural tissue block with an indication of epicardial fibre-orientation (black arrow) and transmural sheet-orientations (green double-lines). The helix-angle (*α*) describes the deviation (viewed from the epicardium) of fibre-orientation from the horizontal plane; it is negative if fibres point from top-left to bottom-right (and positive if they go from bottom-left to top-right). The angle *β* is used to describe the deviation, from the heart's chamber-horizontal plane, of the apparent sheet-orientation in transmural long-cuts of the ventricles, and is positive if sheet-projections are directed in an apico-basal (‘upward’) orientation, as one follows them from endo- to epicardium (and *vice versa*). Also shown is the sheet intersection-angle *δ*, discussed in more detail elsewhere in the text.

Within ventricular myocardium, myocytes are regionally orientated approximately parallel to each other, and the resulting locally prevailing myocyte orientation is referred to as ‘fibre-orientation’. Fibre-orientation was originally studied via histological measurements in sections of transmural ventricular tissue (Streeter et al., 1969; Streeter and Hanna, 1973). It is now understood that fibre-orientation undergoes a transition from a left-handed helical arrangement in sub-epicardial layers, to a circumferential alignment in mid-myocardium, to a right-handed helical direction in sub-endocardial tissue (Jiang et al., 2004). Fibre-orientation is commonly quantified using the helix-angle (*α*, also known as the ‘fibre-angle’), which describes the deviation from the chamber-horizontal plane (defined as perpendicular to the left ventricular long-axis), as viewed from the outside (Fig. 1).

Building on long-established anatomical records, recent studies have confirmed that the ventricular myocardium is further organized in branching sheets (really ‘sheetlets’, as they do not form continuous extended structures that traverse the entire heart), approximately 4–5 cardiomyocytes thick (Costa et al., 1999; Harrington et al., 2005; LeGrice et al., 1995a; Helm et al., 2005a,b). An extracellular collagen-network provides tight coupling of myocytes within a sheet, and looser connections between adjacent sheets that allow for slippage (LeGrice et al., 1995b). Spaces between sheets appear as cleavage-planes in histological sections throughout the ventricles (Costa et al., 1999; Robb and Robb, 1941). They are also apparent from three-dimensional (3D) reconstruction of the ventricular perimysial collagen-network (Pope et al., 2008).

Detailed knowledge of fibre- and sheet-arrangement, and of their changes during the contractile cycle, is essential for understanding cardiac mechanics. Cardiomyocyte contraction provides the cellular basis for force-generation and shortening, predominantly along the fibre-direction. Given the incompressible nature and constant volume of the cytosol, cell shortening is associated with radial widening. Interestingly, the increase in myocyte diameter during contraction would raise wall-thickness by only 8%, while in reality systolic wall-thickening by 40% or more is observed. Thus, cell-diameter increases account for a fraction only (about one fifth) of total ventricular wall-thickening during contraction (LeGrice et al., 1995a,b; Rademakers et al., 1994; Spotnitz et al., 1974). Other contributions are believed to arise from lateral shearing and reorientation of sheets. It has been suggested that, in addition to shearing and reorientation, sheets contribute to systolic wall-thickening by extending the in-sheet plane transverse to fibre-orientation (Spotnitz et al., 1974; LeGrice et al., 1995a,b; Costa et al., 1999).

**Fig. 2:**
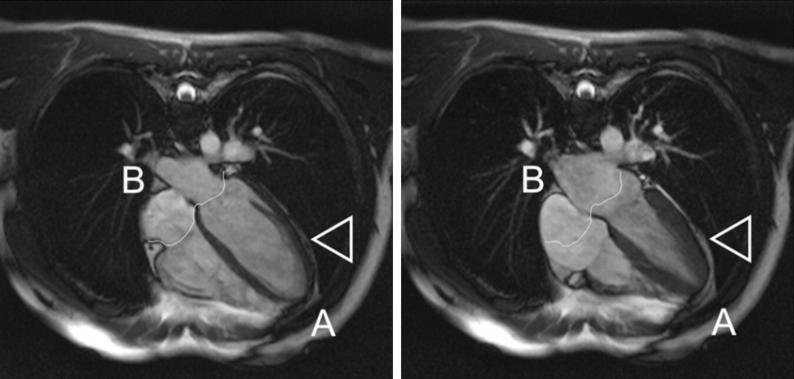
**MRI of a human heart in end-diastole (left) and end-systole (right)**. Note stable position of apex and base (A, B), and minimal lateral translocation of epicardial boundaries (arrow-head). Intra-ventricular volume reduction is dominated by (i) pronounced centripetal tissue thickening, and (ii) the baso-apical shift of the atrio-ventricular valve-plane (see white outline). This is different from the pronounced apex “skipping” seen in isolated coronary-perfused hearts, where the removal of pericardial constraints (which provide a pleura-like viscous coupling that allows for ventricular twisting and sliding, but prevents tissue disconnection) changes cardiac pump function from the normal mode of combined ventricular pressure and atrial suction generation to a ventricular pressure only mode. Modified from (Petersen, 2006); with permission.

In spite of the role sheets are likely to play in the mechanics of cardiac contraction, sheet-architecture and 3D re-organization during systolic wall-thickening are not well-understood (Gilbert et al., 2007). There appear to be two conceptually different models. The first follows the original description of LeGrice et al. (1995a,b), in which smoothly varying sheets radiate outward from sub-endo- to sub-epicardium. The second model allows for the existence of multiple populations of sheets, referred to as alternating sheet-populations/polarities. Early work by Arts et al., 2001 predicted that two distinct populations of sheets should occur, with sheet intersection angles of about 70–90°, based on the hypothesis that sheets are orientated along the plane of maximal systolic shear, and subject to the constraint that muscle fibre axis is fixed in this plane. This was verified experimentally at various locations throughout the myocardium in the canine heart, with the exception of regions close to the epicardium (Arts et al., 2001). Later studies by Ashikaga et al. (2004, 2005) confirmed the existence of two distinct, approximately perpendicular, sheet populations in the canine heart. Histological measurements of sheet orientation in the ovine left ventricle by Harrington et al. (2005) showed an alternating pattern of sheet polarity across the width of the left ventricular wall, suggesting that sheet populations of opposite polarities are present throughout the myocardium. More recently, combined histological and MR-based studies (Gilbert et al., 2012; Kung et al., 2011) have produced strong evidence in support of the ‘two-population’ sheet model, and it was even suggested by Kung et al. (2011) that when only one sheet population is evident in histological sections, this is likely to be a by-product of sample preparation artefacts, obscuring the second sheet population (Kung et al., 2011). However, as with the majority of previous studies, recent work has been limited to snapshots of ventricular histo-anatomy in only one mechanical state, and an understanding of *dynamic* sheet re-arrangement during ventricular contraction is still lacking. Limited insight into sheet-dynamics may be one of the reasons why even the most-advanced histo-anatomically detailed mathematical models of cardiac contraction, while covering the ventricular twisting motion caused by the transmurally varying fibre orientation, tend to under-represent the atrio-ventricular valve plane shift (many models actually show baso-apical lengthening of the ventricles during simulated contraction). Computational heart models also tend to only partially capture the predominantly centripetal (*from “Centrum” [Latin for: the centre] and “petere” [Latin for: to seek/to aim at] – i.e. directed towards the centre*) wall-thickening, which is achieved with relatively stable epicardial outlines (Fig. 2).

Much of the prior research has been based on inherently tissue-destructive techniques, such as serial histological sections. Not only does this tend to limit analysis to small regions of the heart, but identification of true 3D sheet-orientation is problematic using this approach. This is because myocardial laminae are tightly packed in native tissue, and the predominant sheet-orientation can change abruptly in the space of millimetres (Cheng et al., 2005). In addition, combined measurement of sheet- and fibre-orientation is difficult, as fibre-angles will best be identified in tangential sections (since cells are aligned near-parallel to the epicardial surface throughout much of the ventricular wall), while sheets have radially-skewed orientations (Harrington et al., 2005) that are best visualized in transmural cuts.

Over recent years, Diffusion Tensor Imaging (DTI) has been validated as a powerful tool for non-destructive determination of 3D myocardial histo-anatomy (Hsu et al., 1998; Scollan et al., 1998) The arrangement of myocytes along locally aligned tracks gives rise to preferential diffusion of water in that direction. Local water-diffusibility, assessed by DTI, is described by the diffusion ellipsoid, whose long axis (primary eigenvector of the diffusion tensor) is co-aligned with fibre-direction (Basser et al., 1994). This technique can be used, therefore, to non-invasively determine the 3D distribution of locally prevailing cell alignment in tissues such as brain white matter or myocardium. Furthermore, there is good evidence to suggest that, in the heart, the secondary and tertiary eigenvectors of the diffusion tensor (the two shorter axes of the ellipsoid) correlate with the trans-fibre/in-sheet and the trans-fibre/trans-sheet directions, respectively (Scollan et al., 2000; Tseng et al., 2003). DTI therefore offers the potential for a comprehensive assessment of 3D fibre- *and* sheet-architecture throughout the intact ventricles. Due to its non-destructive nature, DTI may be used to measure changes in fibre- and sheet-orientation in the *same* heart, at different stages of the cardiac cycle. This would avoid the impact of histological tissue processing on parameters assessed *in-vitro* (Hales et al., 2011). However, cardiac and respiratory motion, together with limited spatial resolution, low signal-to-noise ratio, and long scan-times, present major technical hurdles for *in-vivo* application of cardiac DTI.

The aim of this study was to measure fibre- and sheet-orientations in one and the same living heart, at two different mechanical states. To overcome the obstacles outlined above, rat hearts were excised and perfused via their coronary vasculature (Langendorff-mode) inside a magnetic resonance imaging (MRI) scanner. DTI experiments were conducted in a slack state (using cardioplegic solution, offering an approximation of tissue structure in diastole), and subsequently during contracture (upon lithium-replacement of sodium in the coronary perfusate, to mimic aspects of mechanical systole). These investigations were complemented with higher-resolution DTI after chemical fixation of hearts, and histological follow-up. As a result, we provide novel evidence for the existence of alternating myocardial sheet-populations in rat ventricles, confirmed using DTI and histological data, and insight into their spatial rearrangement during cardiac contraction. Using DTI, we were able to show that sheet-populations in living myocardium deform in an accordion-like fashion, increasing the inter-sheet-angle during contraction, so that sheets become more chamber-horizontally aligned. These data from two mechanical states in the same heart may help to explain how the combined contribution of fibre-orientation and sheet-re-arrangement helps, during contraction, to shorten baso-apical dimensions of the ventricles, while increasing wall-thickness in a preferentially centripetal direction. These ‘signature-deformations’ of the ventricles *in-vivo* are of key importance for cardiac pump function.

## 2 Materials and Methods

### 2.1 Langendorff perfusion rig

**Fig. 3:**
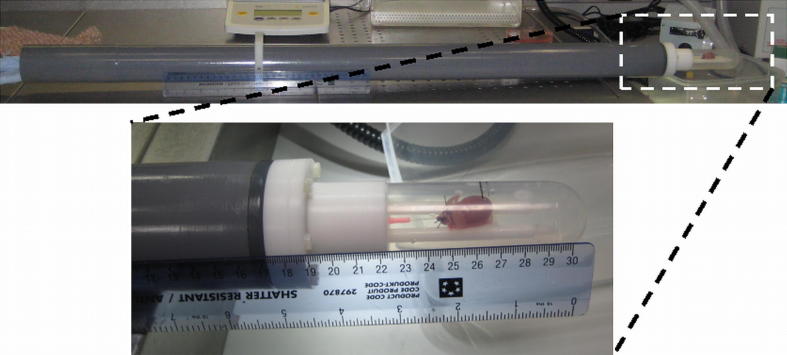
**Photographs of the custom-made Langendorff perfusion rig**. *Top*: view of a heart inside the imaging chamber, mounted to the rigid plastic support tube prior to insertion into the MRI scanner. The perfusate is carried through a water-jacketed line from the reservoir (not shown), through the support tube, and to the Langendorff-mounted heart in the chamber. Effluent solution leaves the system, via gravity, through the support tube. *Bottom*: magnified view of the heart chamber. Hearts were attached to an aortic cannula inside the chamber and placed on a flexible cradle made from Parafilm suspended between two support rods.

A custom-built Langendorff-perfusion rig was used for this work (Fig. 3). The perfusate reservoir was suspended at an elevated level compared to the bore of the magnet, to yield a perfusion pressure of 1.47 × 10^4^ Pa (110 mmHg) and a flow rate of ∼5 ml × min^−1^. All perfusates were oxygenated within the reservoir and gravity-fed into the body of the perfusion rig via flexible water-jacketed Tygon tubing. Incoming and effluent solutions were carried to/from the centre of the magnet inside a rigid plastic support tube, which provided a water-tight compartment to protect the MRI equipment, and facilitated the positioning of heart perfusion chamber at the iso-centre of the magnet and the radio-frequency coil. Following excision (see Section 2.2), hearts were attached to the aortic cannula of the perfusion system, and placed on a cradle made from Parafilm wrap (Cole Palmer, UK) shaped to suit the individual heart. The heart was then enclosed inside the Perspex chamber, attached via a water-tight seal to the rigid plastic tube, and transferred to the bore of the MRI scanner. All component parts were manufactured from plastic/nylon to ensure MRI-compatibility.

### 2.2 Sample preparation

#### 2.2.1 Perfused hearts

All animal work was conducted in accordance with Schedule 1 of the UK Home Office Guidance on the Operation of Animals (Scientific Procedures) Act of 1986, with approval of the Oxford University ethical review board. Hearts were excised from female Sprague–Dawley rats, after concussion of the brain followed by cervical dislocation (*n* = 8, 200–250 g body weight). The aorta was swiftly cannulated for coronary perfusion in Langendorff mode. An incision into the pulmonary artery wall just past the right ventricular outflow valve was performed to prevent intra-ventricular fluid accumulation. After the initial wash using normal Tyrode solution (in [mM]: NaCl 140.0, KCl 5.4, MgCl_2_ 1.0, HEPES 5.0, Glucose 11.0, CaCl_2_ 1.8), cardioplegic arrest was induced using a modified Tyrode solution with elevated K^+^ (in [mM]: NaCl 105.0, KCl 25.0, MgCl_2_ 1.0, HEPES 10.0, Glucose 11.0). Following imaging of the heart in its slack state (see Section 2.3), contracture was induced by switching the perfusate to Na^+^-free Li^+^-Tyrode (in [mM]: LiCl 125.0, KCl 5, MgCl_2_ 1.0, HEPES 10.0, Glucose 11.0, CaCl_2_ 2.5). All solutions were titrated to pH 7.4, and osmotic pressure was checked prior to use with a freezing-point reduction semi-micro osmometer to be within 300 ± 10 mOsm (Knauer, Berlin, Germany). To ensure a complete tissue equilibration in target solutions, measurements were started 20 min after solution switching (room temperature).

#### 2.2.2 Fixed hearts

For high resolution DTI imaging (see Section 2.3.2), hearts (*n* = 2) were briefly washed by Langendorff-perfusion with normal Tyrode, arrested using cardioplegic Tyrode, and fixed in either slack state (high-K^+^) or contracture (high-Li^+^) via coronary perfusion with Karnovsky's fixative (for further detail see (Plank et al., 2009; Karnovsky, 1965)). Fixed hearts were stored in iso-osmotic Karnovsky's (300 mOsm) for 24 h at 4 °C, rinsed in iso-osmotic cacodylate buffer, and then embedded in iso-osmotic 2% low-temperature melting agar for stabilization in the centre of a 28 mm NMR tube.

#### 2.2.3 Histology

For histological verification, two hearts were excised, briefly Langendorff-washed using normal Tyrode, then exposed to either cardioplegic- or Li^+^-Tyrode, and fixed by coronary perfusion with Karnovsky's fixative, as described above. Hearts were stored in Karnovsky's for 48 h, and then wax-embedded. Embedding involved dehydrating the tissue in several steps with ascending concentrations of ethanol, xylene, and finally wax, as described in detail elsewhere (Burton et al., 2006). The histological follow-up involved: serial sectioning of the entire heart, at 10 μm thickness, in a long-axis plane perpendicular to the inter-ventricular septum, Trichrome staining using an automated tissue stainer (AutoStainer XL, Leica, Wetzlar, Germany), and light-microscopic image acquisition using a mosaic workstation (Leica LAS Power Mosaic; further details in (Plank et al., 2009)).

### 2.3 MRI data acquisition

All imaging was performed using an Agilent 9.4 T (400 MHz) MR system (Agilent Technologies, Santa Clara, CA [formerly Varian]), comprising of a horizontal magnet (bore size 210 mm), a VNMRS Direct Drive console, and a shielded gradient system (1 T m^−1^, rise time 130 μs, inner diameter 60 mm). A birdcage coil with an inner diameter of 39 mm (Rapid Biomedical, Rimpar, Germany) was used to transmit/receive the MRI signals for the perfused heart experiments. For high resolution DTI of fixed hearts, a birdcage coil with an inner diameter of 28 mm was used (Rapid Biomedical, Rimpar, Germany).

#### 2.3.1 Sample stability

Initial tissue-stability measurements were made to monitor the effect of interstitial oedema on tissue-architecture during exposure to crystalloid solutions. For this, two hearts were cardioplegically arrested and imaged over a period of 165 min. The time delay between excision and the start of imaging was roughly 15 min. During the imaging time, myocardial T2 values and left ventricular (LV) wall thickness were measured 15 times in an uninterrupted cycle of 11 min intervals, using a 2D multi-spin-echo pulse sequence. Imaging parameters were as follows: number of slices = 5, slice thickness = 1 mm (spaced equally from apex to base), matrix size = 128 × 128, field of view (FOV) = 20 mm × 20 mm, number of averages = 2, TR = 2.5 s. Sixteen echoes per excitation were acquired with TE values equally spaced between 4.6 ms and 73.3 ms.

#### 2.3.2 Diffusion tensor imaging

Following the tissue stability control experiments, DTI-based measurements of myocardial histo-architecture were performed in eight individual hearts, each imaged in both slack state and contracture. Firstly, scout images were acquired to determine short- and long axes of the heart. A multi-slice fast spin-echo sequence with diffusion sensitizing uni-polar gradients was then used to acquire diffusion-weighted images in the short-axis orientation of the heart. Imaging parameters were as follows: TR = 1 s, 8 echoes per acquisition, TE_eff_ = 15 ms (central line of *k* space collected during the first echo), matrix size = 128 × 128, slice thickness = 1 mm, number of slices = 13 (contiguous slices covering the heart from apex to mitral valve), number of averages = 12. The in-plane FOV was adjusted to match the size of each heart, and ranged from 19 to 23 mm, giving an isotropic in-plane resolution of 148–180 μm. Diffusion gradient strength was 31 G cm^−1^, with a gradient duration of 2.5 ms, separation of 9.6 ms, and maximum b-value = 679 s mm^−2^ (including imaging gradients and cross-terms between imaging and diffusion gradients). Ten non-co-linear gradient directions were used, arranged according to an optimized scheme based on the electrostatic repulsion principle (Jones et al., 1999). Total scan time was 32 min, and the above protocol was carried out twice for each heart, i.e. once in slack state and once in contracture.

In order to obtain high resolution DTI data, two further hearts (one slack, one contracture) were chemically fixed, as described in Section 2.2, and a 3D version of the above fast spin echo sequence was used, with the following parameters modified from the 2D sequence: matrix size = 256 × 256 × 256, FOV = 26 mm isotropic, number of averages = 4, scan time = 118 h.

### 2.4 Data analysis

Diffusion tensors were calculated on a voxel-by-voxel basis via a weighted linear least squares fit method, using in-house software developed in IDL (Interactive Data Language, ITT Corporation, CO). Tensors were then diagonalized to produce the sorted eigenvectors (***ν***_1_, ***ν***_2_, ***ν***_3_) and eigenvalues (*λ*_1_, *λ*_2_, *λ*_3_). From these, the Apparent Diffusion Coefficient (ADC) was calculated as the mean of the three eigenvalues, and the Fractional Anisotropy (FA) was determined using the standard method described in Basser et al. (1994).

Eigenvectors were transformed from the global magnet coordinate system into the anatomical coordinate system of the heart using the method described in Geerts et al. (2002), which is based on characteristics of the myofibre field, derived from DTI. Full details of this process are given in Geerts et al. (2002), and briefly explained herein. The first estimate of the local long-axis of the heart was taken as the normal to the imaging plane in the un-weighted (*b*_0_) DTI images. In every slice, voxels within the LV myocardium in which ***ν***_1_ had a minimal out-of-plane component (i.e. approximately circumferentially orientated myofibres) were selected as a first estimate of the mid-wall region. The centre of a best-fit circle through these pixels provided a first estimate of the centre of the LV in each slice. The long-axis direction was then recalculated as the best-fit line through the centres of all these circles, allowing for transformation of the eigenvectors into a new coordinate system defined by the fitted long-axis. The intersection of the derived long-axis with each LV cross section was taken as the new LV centre in each slice. This served as the origin of a local cylindrical coordinate system, and the local radial axis (in the chamber-horizontal plane) was defined as normal to the longitudinal axis. The circumferential direction was defined as normal to the radial axis (i.e. the tangent). As this model represents a simplification of the true underlying geometry, errors may occur in the estimation of the LV long axis. These errors are likely to be greatest near the apex, where local geometry is more spherical, and the local long axis direction is inherently ill-defined. Further away from the apex, Geerts et al. (2002) estimated that the coordinate system used here results in a maximum expected alignment error between the fitted and true LV long axis of approximately 10°, which produces a maximum error in the calculated absolute myofibre orientation of a similar amount. Intra-individual comparisons suffer from a significantly smaller error.

Myofibre orientation was expressed in terms of the helix angle (Streeter et al., 1969), *α*, defined as the angle between (a) the projection of ***ν***_1_ onto the circumferential–longitudinal plane (tangential plane, i.e. parallel to the epicardial surface; see *x*/*y* plane in Fig. 8) and (b) the circumferential–radial (or ‘chamber-horizontal’) plane (see blue dashed in Fig. 1). Helix angles were defined as positive for a right-handed helical orientation (found in the sub-endocardium) and negative for a left-handed helical orientation (found in the sub-epicardium). To aid comparison between datasets, myocardial tissue voxels were binned, according to helix angle, into three types, containing left-handed helical fibres (LHF): *α* < −30°, circumferential fibres (CF): −30° ≤ *α* ≤ 30°, and right-handed helical fibres (RHF): *α* > +30°.

The transverse (or imbrication) angle is defined as the angle between the local circumferential direction and the projection of the fibre direction onto the circumferential–radial plane. As myofibres run near-parallel to the epicardial surface in much of the ventricular tissue, transverse angles are generally small, ranging from −20° to +20° in the rat heart (Chen et al., 2005). As the greatest transverse angles are expected in the most apical slices (Geerts et al., 2002), and since (as described above) determination of the long-axis suffers from the largest inaccuracies in this region, transverse angles were not compared. Of note, it has been shown previously in the rat heart that no significant change occurs in the transverse angle during contraction (Chen et al., 2005).

Sheet orientation was expressed in terms of the sheet angle, *β*, which describes the angle between the heart's chamber-horizontal plane (defined as perpendicular to the LV long axis) and the apparent sheet orientation in a transmural (apico-basal) long-cut (see Fig. 1). Planes are generally defined by the unit vector which is orthogonal to the surface of the plane, and in the case of myolaminae this is the tertiary eigenvector, ***v***_3_. Sheet angles (*β*) were calculated therefore for every voxel by projecting ***v***_3_ onto the voxel-specific longitudinal–radial plane: the angle between this projection and the radial unit vector is equal to (90° – *β*). Note that this method using the tertiary eigenvector is un-ambiguous in terms of defining sheet orientations in a 3D space, and their projection onto the longitudinal–radial ‘cutting plane’. However, when sheet angles are defined using the secondary eigenvector as a substitute, errors can be introduced into sheet angle calculations, as the secondary eigenvector (which runs in the sheet) does not uniquely define the sheet plane in 3D. Sheet angles were defined as positive if the sheet projection in the transmural long-cut moves towards the LV base as one follows the sheet radially from endo- to epi-cardium (Fig. 1). In addition, an automated routine was developed to identify positions throughout the myocardium at which sheet populations of opposite polarity approach one another. At these locations, the angle between the two populations, termed the sheet intersection-angle (*δ*), was calculated as $\delta =180-\left(\left|\beta_{1}\right|+\left|\beta_{2}\right|\right)$, where *β*_1_ and *β*_2_ are the measured sheet angles in each population (see Fig. 1).

Regions of interest (ROIs) were established in each dataset to define apical, equatorial and basal regions of the ventricles (covering approximately one third of the LV base–apex length each), and mean values of helix and sheet angles were calculated as a function of transmural position for each ROI in an individual heart, in slack state and during contracture. In addition to the DTI metrics described above, wall thickness, defined as the mean transmural distance between the LV epi- and endo-cardium in the un-weighted DTI images, was measured along four transmural paths, in the most ‘superior’ (or basal) slice of the equatorial ROI in each heart (i.e. the slice positioned at one-third of the long-axis length from the base of the heart (Streeter et al., 1969)).

Computation of DTI data and analysis, comparing the two mechanical states, were performed using purpose-written software in IDL. Unless otherwise stated, values are expressed as mean ± standard deviation. All data were checked for normality using Anderson–Darling tests, except for sample stability data, in which the Pearson test was used. For normally distributed data, same-organ measurements were compared using a two-tailed paired *t*-test (e.g. to test for overall regional (rather than voxel-wise) changes within one heart, in two mechanical states), inter-individual comparisons used an un-paired two-tailed *t*-test. In cases where the data were non-normally distributed (the fractional fibre orientation data, and sheet-angle histograms before and after contraction), a *G*-test and a two-sample Kolmogorov–Smirnov test were used, respectively, to examine statistical significance of contracture-induced change (see Section 3.3). An ANOVA test was used to check for significant changes in the sample stability data. A value of *p* < 0.05 was considered indicative of statistically significant differences between means.

## 3 Results

### 3.1 Sample stability

In the initial control experiments, hearts scanned at 11 min intervals over a 165 min period showed little sign of gross anatomical change, such as would be indicative of progressive interstitial oedema. Any oedema developing acutely upon exposure to crystalloid perfusion could not be assessed, as scans could be started only at ∼15 min after organ excision, due to preparation time-requirements. The mean myocardial T2 at the beginning of observations and after 160 min remained stable (28.5 ± 1.5 ms *vs*. 28.3 ± 1.9 ms). LV wall-thickness in equatorial slices from the T2 experiments was initially 2.5 ± 0.7 mm, and 2.4 ± 0.6 mm after 160 min. Linear regression of the T2 and wall thickness values, with ANOVA analysis of the slope of the regression line, confirmed that, overall, the samples remain stable over this period.

### 3.2 Global structural changes during contraction of perfused hearts

Mean equatorial LV wall-thickness, mean ADC, and mean FA throughout the myocardial volume before and after induction of contracture are shown in Table 1. During contracture, mean wall-thickness increased by 31 ± 9% (compared to slack hearts; two-tailed *t* test, *p* < 0.001). However, FA and ADC showed no significant difference between slack state and contracture, suggesting that neither ‘cellular density’ nor the intra-voxel *distribution* of fibre-orientations changed significantly on the whole-ventricular scale. In slack state and contracture, the ratio of *λ*_1_/*λ*_2_ was 1.51 ± 0.03 and 1.48 ± 0.07 respectively, and the ratio of *λ*_2_/*λ*_3_ was 1.47 ± 0.04 and 1.43 ± 0.10 respectively.

### 3.3 Regional structural changes during contraction of perfused hearts

**Fig. 4:**
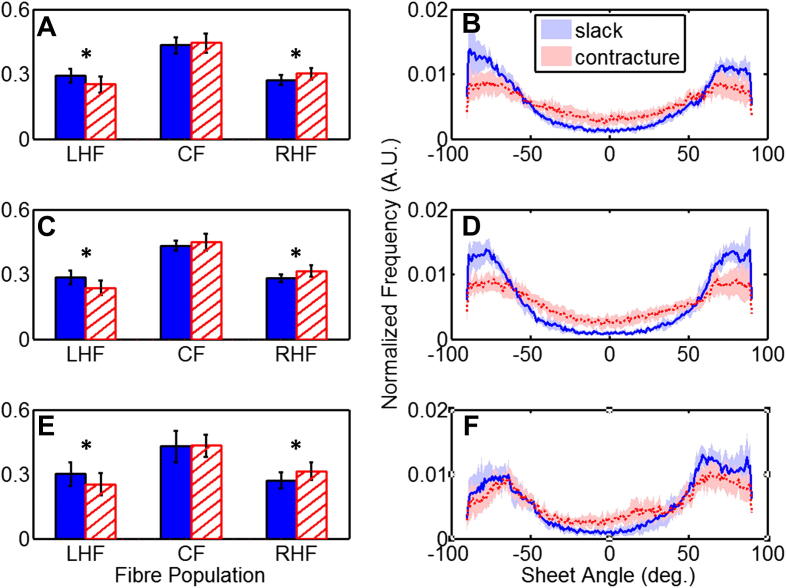
**Summary of changes in helix- (left) and sheet- (right) angles (*n*** = **8 hearts)**, **in basal (top-row), equatorial (middle-row), and apical (bottom-row) regions of the ventricles**. The two states are shown in all panels as blue / solid bars (slack) and red / hatched bars (contracture). A, C and E: Binned fractions of left-handed helical fibres (LHF: *α* < −30°), circumferential fibres (CF: −30° ≤ *α* ≤ 30°), and right-handed helical fibres (RHF: *α* > 30°; all data shown as mean ± SD, *source of significant change in fibre population distribution, after decomposition of *G* statistic by fibre orientation (see Section 3.3)). B, D, and F: Normalized histograms of the distribution of sheet-angles. Solid / dashed lines show the mean values for all slack / contracture hearts studied respectively, while the shaded bands represent the 95% confidence interval for each mechanical state.

**Fig. 5:**
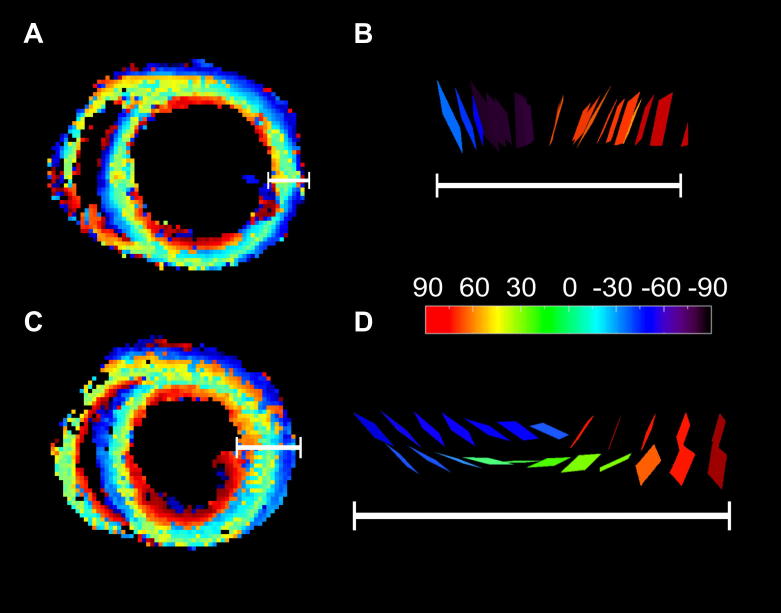
**Plots of helix-angles (left) and sheet-orientation (right), observed in an equatorial layer of one and the same perfused rat heart**, **in slack state (top) and contracture (bottom)**. A, C: helix-angles. B, D: colour-coded rectangles, representing locally-resolved spatial distribution of sheet-orientation (calculated as planes perpendicular to the tertiary eigenvector of the diffusion tensor). Colour-coding applies to all four panels: red = +90°, blue = −90°, see colour bar); white bars represent the location of the sheet-angle profiles and their spatial extent (local LV wall-thickness) relative to the ventricular cross-sections shown on the left.

Contraction-associated changes in fibre-orientation in perfused hearts are shown in Fig. 4 (A, C, and E). During contracture, a consistent decrease was observed in relative proportions of LHF, by 14.0%, 17.3%, 15.8% in the basal, equatorial, and apical sub-epicardium respectively. This was accompanied by an increase in right-handed helical fibres, by 11.0%, 12.1% and 16.1% in the basal, equatorial, and apical regions of the sub-endocardium, respectively. The slack/contracture fibre orientation counts for each part of the heart were analysed using 2 × 3 *G*-tests. All individual tests on 3 locations (basal, equatorial, apical) for *n* = 8 hearts (24 tests in total) were significant at the 10^−6^ level. To aggregate the data across hearts, the contingency tables were merged into a single table per location in the heart. The total fibre counts per sample were normalized, to avoid giving undue weight to samples with a large fibre count overall. Differences were significant for all three cardiac tissue regions: basal (3.68 × 10^−9^), equatorial (1.07 × 10^−129^) and apical (4.84 × 10^−58^). In order to examine the source of the difference, the terms of the *G* statistic were decomposed by orientation. Values for LHF, CF and RHF were (respectively) in the basal region 27.31, 2.65, and 8.88, in the equatorial tissue 403.51, 23.36, and 167.05, and in the apical part 146.98, 0.71, and 116.26. The CF values were smallest throughout, showing that the significant differences (above) are driven predominantly by changes in LHF and RHF values, rather than by a change in the proportion of CF *per se*. An example of changes in helix-angle distribution throughout an axially-orientated slice in one of the perfused hearts, before and during contracture, is illustrated in Fig. 5 (panels A and C).

**Fig. 6:**
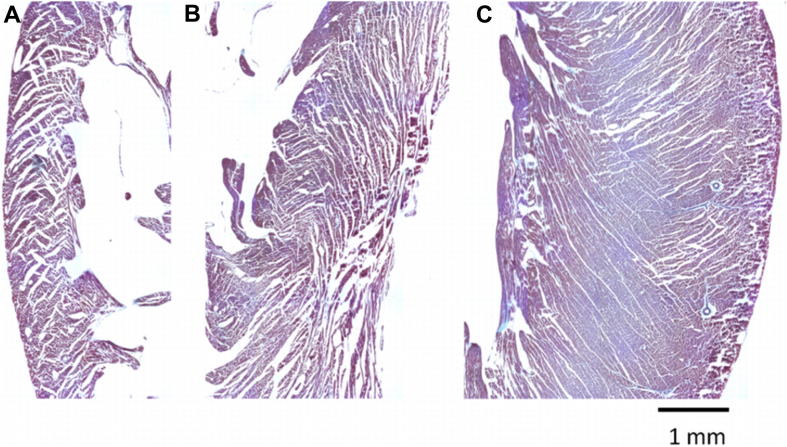
**Long-axis histology sections (apical direction pointing down) of rat myocardium, following tissue fixation in contracture (10 μm section thickness, trichrome stained)**. Cleft spaces with alternating orientation, compatible with multiple transmural sheet populations, can be observed in the right ventricular wall (A), septum (B), and LV wall (C).

Sheet-angle distribution histograms at rest and during contracture are also shown in Fig. 4 (panels B, D, and F). A bimodal distribution of sheet-angles was observed in all hearts, in both mechanical states. The two main populations had sheet-angles of opposite polarity, referred to henceforth as the *β*+ and *β*− populations (see Figs. 1 and 6 for a histological illustration). Using the same criteria employed to compare group helix-angles, sheet-angles were classified as belonging to the *β*− population for *β* < −30°, and the *β*+ population for *β* > +30°. A third group, with sheet-angle values centred around 0° (*β*_0_), included the range −30° ≤ *β* ≤ 30°. A two-sample Kolmogorov–Smirnov test confirmed that the distribution of sheet-angles changed significantly (*p* < 0.001) between the two mechanical states, in basal, equatorial, and apical regions of the hearts. The greatest shift in sheet-orientation was observed in the equatorial region, where the absolute values (|*β*|) of the mean sheet-angle in the *β*+ population decreased by 8% (from 69.3° to 64.1°, *p* < 0.01), and in the *β*− population by 8% (from |−69.3|° to |−63.7|°, *p* < 0.01). A shift of similar magnitude was observed in the basal region, with a decrease in *β*+ by 6% (from 67.0° to 62.9°, *p* < 0.01), and in *β*− by 7% (from |68.3|° to |−63.6|°, *p* < 0.01). A smaller shift occurred in the apical region, where the only significant change was a 4% decrease in the absolute *β*− sheet-angle (from |−65.4°| to |−63.0°|, *p* < 0.01). The 95% confidence intervals of sheet-angle distributions, illustrated in panels B, D, and F of Fig. 4, highlight the separation between contracture and slack state, in particular in equatorial and basal layers. The 95% confidence intervals of sheet-angle distributions, illustrated in panels B, D, and F of Fig. 4, highlight the separation between contracture and slack state, in particular in equatorial and basal layers.

The re-alignment of sheets into more horizontal planes during systole can make a significant contribution to wall thickening. Table 2 illustrates this by comparing mean sheet-angles encountered in the bottom-, middle- and top-third percentiles of measured values, in slack state and contracture. Theoretically predicted segmental contributions to transmural wall thickening range from 16% (apical third of the ventricles) to 37% (equatorial region; see Table 2 and Discussion). This is also apparent from the data presented in Fig. 5 (B, D), where changes in mechanical state are shown to be associated with an accordion-like rearrangement of sheets: contraction is accompanied by a reduction in the absolute magnitude of sheet-angles (note how the more-horizontal alignment of sheets is associated with increased wall-segment width in the same sample). Accordingly, the ‘sheet intersection-angle’ between opposite sheet-populations increases (*δ* in Fig. 1). The mean value of all sheet intersection-angles was 86 ± 6° in slack LV; this increased to 108 ± 5° during contracture (two tailed *t*-test, *p* < 0.001). Regionally, increases in mean sheet intersection-angle of 23%, 29% and 26% were observed in the basal, equatorial and apical parts of the hearts, respectively (two tailed *t*-test, *p* < 0.005 in each region).

**Fig. 7:**
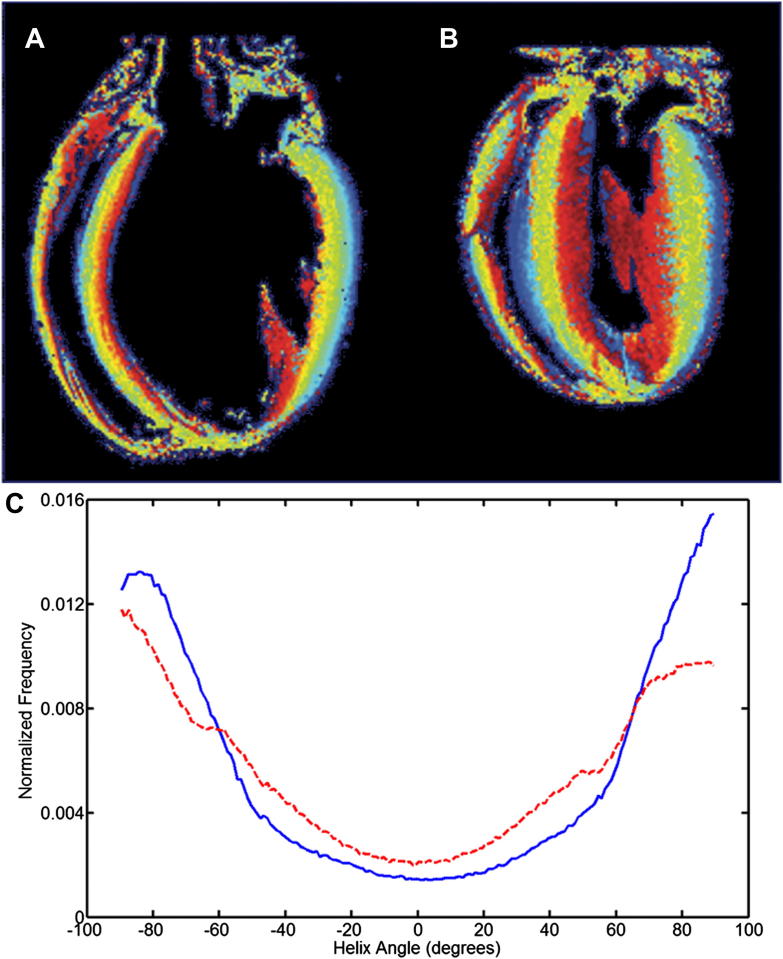
**High resolution DTI**. A, B: Plot of helix angles derived from the high resolution DTI datasets in two hearts, chemically fixed in either slack (A) or contracture (B) states (red = +90°, blue = −90°). C: Normalized histograms of the distribution of sheet angles throughout the myocardium in the above slack (blue/solid line) and contracture (red/dashed line) hearts, illustrating that in contracture the fraction of sheet angles with low absolute values is higher than in the slack state.

The presence of intersecting sheet-patterns was reconfirmed using high-resolution DTI of fixed myocardium, and histology. Histograms of sheet-angle distribution in the entire myocardial volume, derived from high-resolution DTI data in slack state and contracture, are shown in Fig. 7. In the slack heart, the majority of sheet-angles lie in either the *β*+ or *β*− populations, and the relative proportion of sheet-angles that were orientated more radially (in the range 30° < *β* < 30°) was only ∼11%. In contracture, this fraction of sheet-angles outside of the dominant *β*+ or *β*− populations increased to 15%.

Histological assessment re-confirmed the presence of regions where opposing sheet-populations meet in mid-myocardial layers of right ventricle (RV), LV, and septum (Fig. 6). However, since histological images and high-resolution DTI from fixed tissue offer information only for a single mechanical state in any given sample, it is not possible to comment in these datasets on transitions of *β*+ and *β*− sheet-populations during ventricular deformation. Statistical comparison of changes in sheet- and fibre-angles was restricted, therefore, to same-heart DTI data from live-perfused organs.

## 4 Discussion

The aim of this study was to measure regional changes in fibre- and sheet-architecture in different mechanical states in one and the same sample, using DTI of living heart tissue. During the time required for DTI (64 min), hearts offer a histo-anatomically stable substrate. According to our sample-stability assessment, there is no significant build-up of oedema, beyond what will have occurred immediately after cardiac excision and the onset of saline perfusion (Bub et al., 2010), over a period of more than 2.5 h. Our sample stability measurements also confirm that no unwanted contracture (before perfusion with lithium) occurs in the cardioplegically arrested hearts.

A previous study by Chen et al. (2005) compared helix- /sheet-angles in live-perfused slack and contracture-fixed rat hearts. In contracture, they found increased helix-angles for both LHF and RHF populations, and more radially-aligned sheets. However, as changes in absolute sheet angles were compared (essentially treating sheets as one population), sheet intersection-angles were not analysed. Also, tissue in contracture was chemically fixed prior to imaging, which alters histo-anatomical structure of rat ventricular tissue (Hales et al., 2011). To the best of our knowledge, this is the first study performing DTI on the same live-perfused heart in two different mechanical states. Contrary to Chen et al. (2005), during contracture we see a *decrease* in sub-epicardial LHF and an accompanying increase in sub-endocardial RHF throughout the myocardium. The latter may be caused in part by the fact that there is a centripetal translocation of myocardial tissue mass, which is associated with a reduction in the mean radii of tissue layers. This is the more pronounced the further away tissue locations are from the epicardium. During contraction, the RHF-containing sub-endocardial tissue will therefore occupy a larger fraction of the ventricular cross-section, compared to the resting state. In addition, it is possible that endocardial tissue, including trabaeculations, becomes compacted during contraction, so that fibres that may not be traceable by DTI in the slack state become apparent in contracture.

Our data further show that sheet-populations with alternating polarity, forming V-, X-, N-, W- or M-like shapes in transmural long-cuts, are present throughout the living myocardium, and that the intersection-angle between these populations increases during contraction. This change in sheet intersection-angles is caused by a dynamic re-organization of ventricular tissue, which is most apparent in the basal and equatorial regions of the heart. Higher resolution DTI data, taken from fixed hearts, re-confirm the existence of alternating sheet-populations, and (although not based on paired observations) they also indicate an increase in sheet intersection-angles during cardiac contraction.

Similar patterns of alternating sheet-orientations can be detected (but are not always mentioned) in previous DTI (Jiang et al., 2004; Helm et al., 2005a,b; Chen et al., 2005) and high-resolution confocal microscopy studies of LV tissue from rats (Sands et al., 2005). Harrington et al. (2005) measured sheet-angles in slack-fixed sheep hearts, using histological methods, and observed two populations of sheets, clustered around +45° and −45°, which agrees well with our mean sheet-intersection-angle of 86.2° in the slack state. Our data also show excellent agreement with the histologically measured sheet intersection-angle in the end-diastolic state in the study by Kung et al. (2011). In Harrington's paper, the authors describe an accordion-like organization of myocardial layers, in which alternating sheet-families are observed when traversing the ventricular wall. It was speculated that wall-thickening could be a result of increased intersection-angles between the two sheet-populations, an idea whose origin can be traced to anatomists of the early 20th century. Due to the tissue-destructive nature of prior measurements, this could not be verified before. We now demonstrate, within one and the same individual heart, that re-arrangement of alternating sheet-populations does indeed occur during transition from the slack state to contracture, and that this is associated with an increase in sheet intersection-angles.

**Fig. 8:**
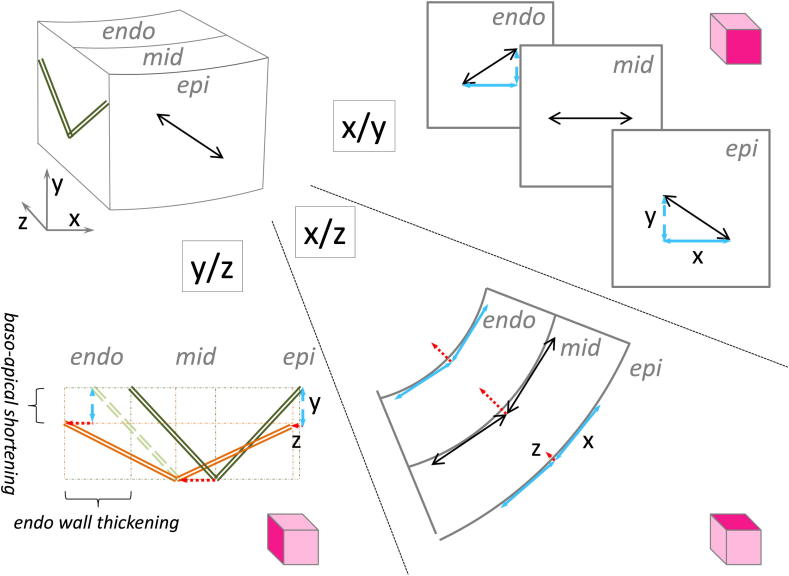
Schematic illustration of the proposed interaction between fibre- and sheet-orientations, and how their interplay may contribute to wall-thickening and apico-basal shortening. Clockwise, starting top-left: illustration of a transmural tissue-block, removed from LV free-wall (as in Fig. 1), with introduction of the *x*/*y*/*z* coordinate system used in this illustration. Top-Right (*x*/*y*): epicardial view onto different layers of the tissue block, illustrating decomposition of forces in *x* and *y* directions. Bottom-Right (*x*/*z*): top–down view onto the same tissue block (transmural/horizontal plane) with indication of how curvature of ventricular wall-segments affects decomposition of tangential (here taken to equal the *x*-component) into centripetal forces (*z*-direction). Bottom-Left (*y*/*z*): side-on view of the tissue-block (transmural/baso-apical plane), with indication of sheet-angle re-arrangement from rest (green) to contraction (orange) by applying *y*- and *z*-components established above. Please see more detailed explanation in the Discussion section. Illustration is a highly-simplified scheme and not to scale. Epi: sub-epicardial; mid: mid-myocardial; endo: sub-endocardial layers.

A conceptual model of how the helical arrangement of the myofibres and the accordion-like orientation of sheets could explain (a) the predominantly centripetal displacement of ventricular muscle mass, (b) the absence of major epicardial contour displacements, and (c) ventricular shortening in the ‘longitudinal’ (apico-basal) direction, is illustrated in Fig. 8. The top-left panel is equivalent to the schematic illustration of the excised tissue block in Fig. 1, and introduces the *x*/*y*/*z* coordinates used in the following. Moving clock-wise, the top-right panel identifies fibre-orientation in the *x*/*y* plane (defined as parallel to the epicardial surface, and simplified to ignore curvature, with *x* in the heart's chamber-horizontal and *y* in the apico-basal direction) for a representation of sub-epicardial, mid-myocardial and sub-endocardial layers. Mechanical activity is assumed to be maximal in the fibre-direction (black large arrows). This is decomposed into components in *x* (blue solid arrows) and *y*-directions (blue dashed arrows). Mid-myocardial fibres lie along the *x*-direction and, hence, have a horizontal force component only (*y* = 0). One can now carry the *x*-component of mechanical activity over to the *x*/*z* plane, shown in the bottom-right panel, and plot this in a tangential manner onto epi-, mid- and endocardial layer representations, shown with their different radii. This allows one to visualize that the centripetal component (*z*-direction; red dotted arrows) will be smallest at epicardial locations. Moving towards the mid- and endocardial layers, the reduced radius will favour *z*-translation of mechanical activity in the *x*-direction (this is borrowed from depictions of how surface tension translates into pressure in a hollow sphere, according to the law of Laplace). Since the projection is ‘cut’ in the horizontal plane, the tangential force component that is shown is largest in the mid layer (where fibres are aligned in the same direction as the projection's cut). Note, though, that curvature-based translation of tangential into centripetal forces occurs also in other planes (not shown here for simplicity, but any such translation will always be favoured as one moves from epi- towards endocardium by the reduced radius of local curvature). Now that we have a conceptual illustration of relative mechanical driving forces for deformation in all three directions (*x*/*y*/*z*), we can consider how this could affect a simplified version of sheet populations (only two shown) in the *y*/*z*-plane (bottom left of Fig. 8). The transition from the relaxed state (sheets drawn in green) to contraction (sheets in orange) is driven here simply by application of relative *Y* and *Z* component amplitudes (established above) to epi-, mid- and endocardial layers. These transitions result in baso-apical shortening of the ventricular block model, and wall thickening predominantly in the endocardial direction. These changes arise simply as a result of activation along fibres with transmurally varying angle (initial black arrows; see Fig. 8) and interaction with laterally-reinforced sheetlets that can change their intersection angle.

This schematic illustration is focussed on conceptualizing a small sub-set only of the mechanical re-arrangements, and it is highly simplified. Thus, it doesn't address integrated 3D effects, ignores the presence of multiple sheet-populations across the wall in many regions, excludes chamber-twisting, and it is mechanically ‘imprecise’ (as distinctions between, stress, strain, and consequences of fibre-normal effects of contraction are not distinguished). Yet, it may serve as an illustration of how the presence of sheets, and the increase in their intersection angles during cardiac contraction, may contribute to those changes in ventricular shape that are not well-captured by current conceptual and computational models. The ball-park of measured (25–33%; Table 1) and theoretically-predicted wall-thickening (based on this conceptual approach: 16–37%; Table 2) show considerable overlap. In addition, the proposed schematic may perhaps also offer an indication, on the simplest possible level, of how transmural changes in fibre orientation may drive sheet re-arrangement.

### 4.1 Study limitations

One of the key limitations to our study is that, with a DTI slice-thickness of 1 mm compared to an average sheet-thickness of ∼50 μm (LeGrice et al., 1995a,b), measurements of sheet-angles represent means of at least 20 ‘stacked’ tissue layers, so finer detail is lost. Also, to allow subsequent scans of the same heart, a diffusion imaging scheme with only 10 encoding directions was used. A higher number of gradient encoding directions would increase the accuracy of the diffusion tensor, albeit at the cost of increased scan time. However, the ratio of *λ*_2_/*λ*_3_ was only 3% lower than the ratio of *λ*_1_/*λ*_2_ in our study, which suggests only a small sorting error for the secondary and tertiary eigenvectors. Furthermore, the use of cardioplegic solutions to induce arrest, and of Li^+^ to provoke contracture, will offer approximations only of normal slack and end-systolic states. However, this was required to accommodate the relatively long DTI scan-times in the absence of tissue fixation (which would have defied the purpose of this study). Lastly, the terminology used in the majority of studies in this area may be misleading. The term ‘fibres’ refers purely to the locally prevailing cell-orientation. The term ‘sheets’ describes laterally reinforced cell layers that are linked locally, largely through perimysial collagen, forming local ‘sheetlets’ rather than continuous planes.

## 5 Conclusion

We used DTI to measure regional changes in fibre- and sheet-orientation in living hearts at rest and during contracture. We observed significant changes in the balance of LHF and RHF during contracture, which colloquially could be termed ‘sub-endocardial tissue gain’, which is in keeping with the mainly centripetal direction of ventricular wall thickening. In addition, both DTI and histology data provide strong evidence not only for the *existence* of multiple alternating sheet-populations throughout the ventricular myocardium, but also for their dynamic re-arrangement during contraction into more chamber-horizontal planes. The associated increase in sheet intersection-angles may contribute both to the predominantly centripetal ventricular wall-thickening and to the baso-apical shift in the atrio-ventricular valve-plane *in vivo*. A thorough understanding of the 3D reorganization of fibre- and sheet-structures in the heart is vital for accurate modelling, whether conceptual or computational, of electrical and mechanical behaviour of the heart. This is relevant for the interpretation and optimization of clinical observations, such as related to wall-thickening or ejection-fraction, for example in sinus node *vs*. ventricularly paced beats. Further work in this area, particularly at higher spatial resolution, in mechanical states representative of more points of the cardiac cycle, and including disease conditions with local (e.g. scarring) or diffuse (e.g. fibrosis) alterations in tissue integrity, is likely to lead to a significant improvement in our understanding of cardiac electro-mechanical structure–function relations.

## Editors' note

Please see also related communications in this issue by Markhasin et al. (2012) and Hermeling et al. (2012).

## Figures and Tables

**Table 1:** Mean change in wall LV wall-thickness, FA and ADC.

Parameter	Slack	Contracture	Change (by [%])
Wall-thickness (mm)			
Base	1.68 ± 0.24	2.23 ± 0.44	33*
Equator	1.74 ± 0.25	2.17 ± 0.37	25^†^
Apex	1.48 ± 0.12	1.85 ± 0.37	26^†^
FA	0.36 ± 0.02	0.34 ± 0.04	−6
ADC (×10^−3^, mm^2^ × s^−1^)	1.05 ± 0.08	1.01 ± 0.07	−4

Mean left-ventricular (LV) wall-thickness, fractional anisotropy (FA), and apparent diffusion coefficient (ADC) for hearts in slack and contracture states. Data represent mean ± SD for all hearts studied (*n* = 8). ^†^*p* < 0.05, *p < 0.01, two-tailed *t*-test.

**Table 2:** Measured change in sheet-angle distribution, and theoretical contribution to systolic changes in wall-thickness.

		Measured sheet-angles in		Theoretical contribution to systolic changes in transmural segment thickness (increase to [%])	
		Slack	Contracture	Per sheet-population	Per region
Base	Min-3rd	−76.3 ± 2.6	−68.9 ± 3.7	152%	131%
	Mid-3rd	−6.3 ± 5.9	−1.3 ± 3.6	1%	
	Max-3rd	73.8 ± 1.7	67.2 ± 5.2	139%	
Equator	Min-3rd	−76.6 ± 2.0	−69.7 ± 3.1	150%	137%
	Mid-3rd	1.0 ± 8.1	−2.2 ± 8.2	0%	
	Max-3rd	76.8 ± 2.1	68.5 ± 6.0	161%	
Apex	Min-3rd	−68.3 ± 5.9	−65.1 ± 5.9	113%	116%
	Mid-3rd	24.8 ± 6.7	13.0 ± 5.7	7%	
	Max-3rd	74.6 ± 3.5	70.5 ± 3.6	126%	

Measured sheet-angle values in slack and contracture states are split into bottom-, middle- and top-third percentiles (min-3rd, mid-3rd, max-3rd; respectively), encountered in basal, equatorial and apical layers of the myocardium (*n* = 8 hearts). The theoretical contribution of sheet re-alignment to changes in wall-thickness is calculated assuming a cylindrical model of the ventricular wall where: $\cos{\left|\beta \right|}_{\text{contracture}}/\cos{\left|\beta \right|}_{\text{slack}}\times 100$ = % segment thickness of the contracture *vs*. slack tissue. ‘Per sheet-population’ gives values for each line; ‘per region’ data is the average theoretically predicted wall thickening for basal, equatorial, and apical layers, using an equal weighting of all percentile groups.
